# Practical Notes on Diagnosis and Treatment

**Published:** 1907-07-27

**Authors:** 


### Practical Notes on Diagnosis and Treatment.

### Vomiting of Pregnancy.

Dr. Goodell recommends the following:—Cerium oxalate gr. j., ipecacuanha gr. j., creasote ♏︎ij. Make into a pill; one to be taken every hour until nausea is controlled.

### Pruritus Vulvæ at Menopause.

The following lotion may be applied to the affected parts after ablution with warm water and Castile soap:—Morphine sulphate 6 grains, boric acid 90 grains, camphor water 6 fluid ounces.

### Chordee.

In severe cases opium is needed, and it may be prescribed as a suppository as follows:—Opium in powder 6 grains, camphor 18 grains, cacao butter q.s. Make into six suppositories. Order one at bed-time, and repeat as may be necessary.

### Ocular Paralysis in Tabes Dorsalis.

Diplopia due to an ocular paralysis may be the first symptom of tabes dorsalis. The paralysis is often transitory, but may recur. Hence an ocular paralysis in a patient who has reached middle life, unless dependent on some other obvious cause, is an event highly suggestive of commencing degenerative changes in the central nervous system.

### Abrasion or Ulcer of Cornea.

To bring an abrasion or ulcer of the cornea clearly into view, instil a fraction of a drop of a 2-per-cent. solution of fluorescin; wash off excess with water, and it will be found that wherever the corneal epithelium is absent the part is stained green.

### Brine-Pack.

Flannel wrung out of a saturated solution of salt is a useful application to chronic rheumatoid joints. It should be covered with a layer of dry flannel or with gutta-percha tissue, and may be worn all night.

### Treatment of Pneumothorax.

Morphine or other sedatives may be needed to relieve pain, and sometimes even chloroform may be necessary. Stimulants, too, are usually required. Cupping the chest, or even venesection, is particularly useful when there is much cyanosis. The removal of the air is needed when there is great distension and consequent dyspnœa. A fine trocar should be introduced, and the air be allowed to escape. Anything in the shape of aspiration would probably re-open the wound in the pleura, and more air would thus pass into the pleural cavity.

